# Metabolic insights and novel risk score for adherent perinephric fat in partial nephrectomy: results from a prospective study

**DOI:** 10.1007/s11255-026-05031-5

**Published:** 2026-02-06

**Authors:** Łukasz Mielczarek, Omar Tayara, Wojciech Malewski, Przemysław Szostek, Paweł Rajwa, Riccardo Bertolo, Fabio Zattoni, Carlo Prevato, Sławomir Poletajew, Łukasz Nyk, Piotr Kryst

**Affiliations:** 1 Department of Urooncology and Minimally Invasive Urology, Centre of Postgraduate Medical Education, Warsaw, Poland; 2 Urology Department, Azienda Ospedaliera Universitaria Integrata, University of Verona, Verona, Italy; 3 Department of Surgery, Oncology, and Gastroenterology, Urology Clinic, University of Padua, Padua, Italy

**Keywords:** Adherent perinephric fat, Partial nephrectomy, Renal cell carcinoma, Nephrometry score

## Abstract

**Purpose:**

This study aimed to identify preoperative metabolic and radiological predictors of adherent perinephric fat (APF) and to develop a predictive scoring system for its assessment.

**Methods:**

We conducted a prospective study of consecutive patients with renal tumors undergoing open or minimally invasive partial nephrectomy (PN). APF was intraoperatively defined as the need for subcapsular renal dissection to isolate the tumor. Patient characteristics were compared according to APF presence. Multivariable logistic regression analysis was performed, and the resulting model was used to develop a predictive scoring system.

**Results:**

A total of 200 patients were included in the analysis, of whom 34 (17%) had APF. On multivariable analysis, presence of perinephric fat stranding (*p* = 0.003), posterior perinephric fat thickness ≥ 25 mm (*p* < 0.001), serum urea ≥ 33 mg/dl (*p* = 0.004), albumin ≤ 4.3 g/dl (*p* = 0.007), and HDL cholesterol ≤ 53 mg/dl (*p* = 0.019) were predictors of APF. A model incorporating these five variables achieved an area under the receiver operating characteristic curve of 0.92. These parameters were subsequently integrated into the novel SHARP-U (Stranding, HDL cholesterol, Albumin, Renal Perinephric fat thickness, Urea) score, ranging from 0 to 7, to predict the presence of APF.

**Conclusion:**

The SHARP-U score provides a simple and reliable tool for preoperative prediction of APF in patients undergoing partial nephrectomy. Early identification of individuals at risk may aid surgical planning and patient counseling. External prospective validation of the SHARP-U score is warranted to confirm its clinical applicability.

**Supplementary Information:**

The online version contains supplementary material available at 10.1007/s11255-026-05031-5.

## Introduction

The difficulty in dissecting perinephric tissue remains a well-recognized challenge among surgeons performing partial nephrectomies (PN) [1] and living donor nephrectomies [2]. This phenomenon, often colloquially termed “toxic fat”, is described in the literature as adherent perinephric fat (APF). The presence of APF can increase the technical complexity of surgery by limiting kidney mobilization, impeding the identification of anatomical structures, and hindering the visualization of renal tumors. A standardized and objective definition of APF remains lacking, which is reflected in the wide variability in reported incidence rates, ranging from 10.6% to 55.2% [3, 4].

In a meta-analysis, older age, higher body mass index (BMI), male sex, history of hypertension, and larger tumor size were identified as risk factors for APF [5]. The presence of APF has been linked to adverse perioperative outcomes such as increased blood loss, higher transfusion rates and prolonged operative time [5]. Chronic systemic inflammation, often associated with metabolic syndrome, has been proposed as a potential underlying mechanism of APF, although its precise etiology remains unclear. One study evaluated inflammatory indices derived from complete blood count parameters and identified the systemic immune inflammation index as an independent predictor of APF [6]. In other series, metabolic syndrome was the only independent predictor of APF [7]. Several predictive scoring systems have been developed to estimate the likelihood of APF, most notably the Mayo Adhesive Probability score, originally proposed by Davidiuk et al. [8], and its modified version introduced by Borregales et al. [9], both of which rely solely on radiological and clinical parameters.

To further elucidate the potential contributors to APF, we aimed to assess the association between clinical and radiological characteristics, blood-based metabolic and inflammatory markers, and the occurrence of APF. Based on these findings, we developed a novel preoperative scoring system incorporating both radiological and blood-derived parameters to predict the presence of APF.

## Methods

### Study design

This was a prospective observational cohort study that evaluated patients with a radiologically confirmed renal tumor referred for surgical treatment with partial nephrectomy. The study underwent Ethics Committee approval and was registered in the ClinicalTrials.gov database (NCT05887245). The study was conducted in a single high-volume referral center performing > 150 cases per year. Patients were recruited between April 2023 and December 2024. The prespecified cohort size was 200 patients eligible for full analysis. Consecutive patients qualified for surgery based on radiological diagnosis of kidney tumor were prospectively identified and offered participation in the study. Inclusion criteria included: (1) patients qualified for open, laparoscopic, or robotic partial nephrectomy; (2) tumor stage cT1a-b according to TNM classification; (3) age ≥ 18 years; (4) preoperative contrast-enhanced computed tomography (CT) available for assessment; (5) written consent to participate in the study. Study exclusion criteria were as follows: (1) recurrent ipsilateral renal cancer or prior ipsilateral renal surgery; (2) missing medical documentation that prevents data analysis. After informed consent, 30 ml of venous blood was collected one day prior to surgery. Blood samples were sent to the laboratory, where the following parameters were determined: albumin; total protein; alanine aminotransferase (ALT); aspartate transaminase (AST); gamma-glutamyltransferase (GGTP); alkaline phosphatase (ALP); lactate dehydrogenase (LDH); bilirubin; C-reactive protein; total cholesterol (TC); triglycerides (TG); high density lipoprotein cholesterol (HDL-C); low density lipoprotein cholesterol (LDL-C); ferritin; fasting glucose; creatinine; estimated glomerular filtration rate (eGFR) calculated with the Modification of Diet in Renal Disease equation; urea; total blood count. Anthropometric (age, weight, height, waist circumference) and comorbidity data (assessed with Charlson Comorbidity Index) were collected at the time of enrollment. Metabolic syndrome was defined according to Harmonizing the Metabolic Syndrome: A Joint Interim Statement definition [10] with the International Diabetes Federation thresholds for abdominal obesity. Dyslipidemia was defined as increased levels of serum TC, LDL-C, TG, or a decreased serum HDL-C concentration, or use of hypolipidemic agents.

### Surgical treatment

The patients underwent open, laparoscopic, or robotic PN procedures. The surgical approach was determined based on both the surgeon’s expertise and the patient’s preference. Surgeries were performed by three experienced surgeons, each surgeon performing > 50 PNs annually. The surgeons could review the CT scans and laboratory results preoperatively but were blinded to any APF predictive scores. Open PN was performed retroperitoneally via lumbotomy by Surgeon 1 (PSz). Laparoscopic and robotic PN were performed through the transperitoneal approach by Surgeon 2 and 3 (OT and WM). APF was defined intraoperatively by the surgeon as the presence of “perinephric fat adherent to the renal parenchyma, requiring subcapsular dissection for exposure of the renal tumor”. This definition was agreed upon by all participating surgeons prior to study initiation and applied consistently during PN. Each surgeon filled out a survey after the procedure where APF status was noted.

### Tumor and perinephric fat characteristics

**Fig. 1:**
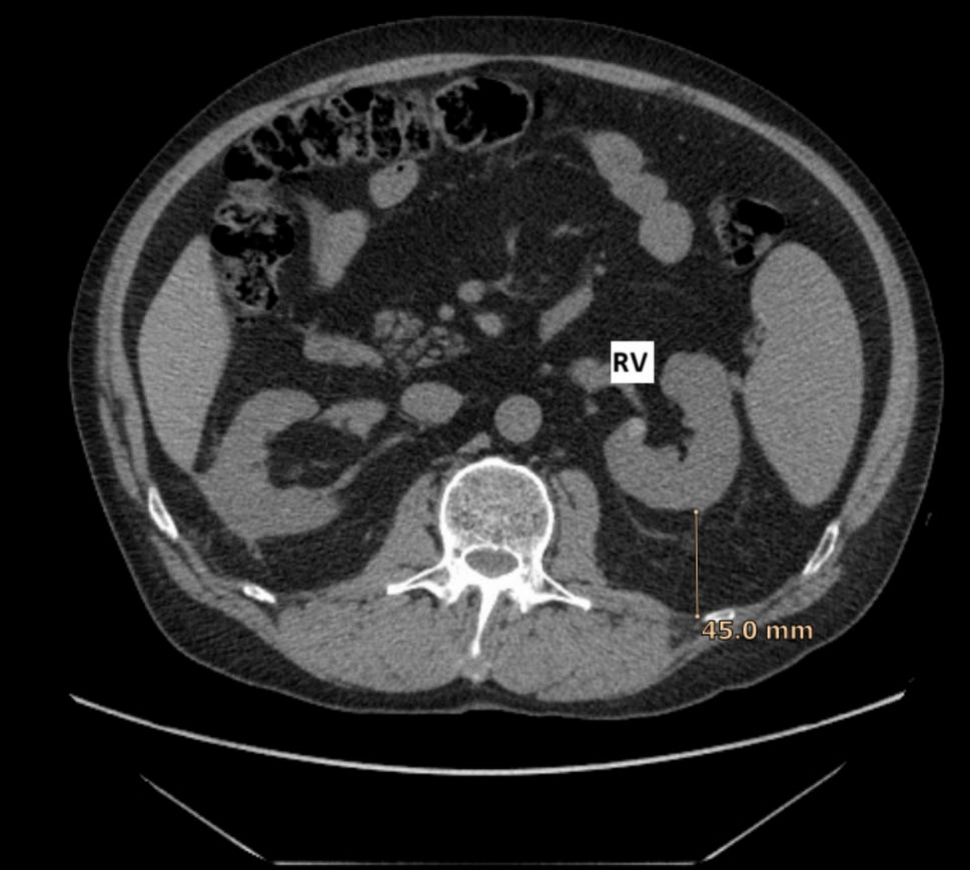
Measurement of posterior perinephric fat thickness as the distance between the posterior renal capsule to the posterior abdominal wall musculature at the level of the renal vein (RV)

**Fig. 2:**
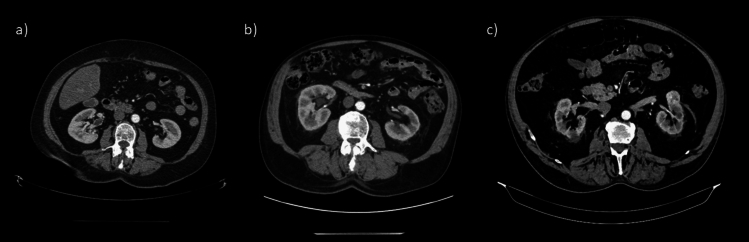
Grading of perinephric fat stranding. **a** absent stranding – no strands are visible; **b** mild/type 1—few thin strands are visible; **c** severe/type 2—many thick strands are visible. Both type 1 and 2 are categorized as present stranding

All patients underwent preoperative chest, abdomen, and pelvis contrast-enhanced CT scans. Tumor characteristics, RENAL nephrometry score, perinephric fat thickness, and the degree of perinephric fat stranding were determined by a junior urologist with dedicated training in urologic imaging, using a standardized evaluation protocol based on previously published criteria. The reader was blinded to intraoperative findings and postoperative outcomes, including the presence of adherent perinephric fat, at the time of CT assessment. Imaging evaluation was performed as part of preoperative planning and was not informed by laboratory results or the study hypothesis. Posterior perinephric fat thickness was measured on axial images at the level of the renal vein as the distance between the posterior renal capsule to the posterior abdominal wall musculature, as described by Eisner et.al [11] and presented in Fig. 1. Perinephric fat stranding was defined as linear areas of soft tissue attenuation in the perinephric space according to Kim et al. [12]. Cases without fat stranding were categorized as none; cases with fat stranding were categorized as mild/type 1 when a few thin strands were visible; severe/type 2 when many thick strands were visible as in Davidiuk et al. [8]. Examples of perinephric fat stranding grades are presented in Fig. 2. Pathological tumor characteristics were reviewed by a genitourinary pathologist, histology was annotated according to the 2016 World Health Organization classification of urogenital tumors [13].

### Statistical analysis

Results are presented as the number and percentage for categorical variables and medians with interquartile range (IQR) for continuous variables. Differences between groups were tested with the Chi-square test for categorical variables and the Mann–Whitney *U* test for continuous variables. Continuous variables significantly different between groups were dichotomized using data-driven cut-offs derived from receiver operating curve and Youden’s index analysis to optimize discrimination for APF presence and to enable construction of a simplified and clinically practical risk score. Univariate logistic regression was used to identify factors associated with APF. Variables that were statistically significant (*p* < 0.05) in the univariate analyses were included in a multivariable model. Stepwise model selection was performed until all variables included in the multivariable model were significant at the 0.05 level. Model discrimination was assessed by calculating the area under the receiver operating characteristics curve (AUC). Internal validation was performed using non-parametric bootstrap resampling (1000 iterations) to estimate model optimism and obtain an optimism-corrected AUC. In addition, a sensitivity analysis was performed using continuous predictors in the model to assess the robustness of the associations. A scoring system was created by assigning points based on the magnitude of each covariate’s odds ratio (OR). To provide more accurate prediction estimates in categories with small sample size, a logistic regression model was constructed using the diagnostic score (range 0–7) as the sole predictor of APF. Sensitivity analyses were performed by re-fitting the primary model with additional adjustment for surgical approach (open, laparoscopic, robotic) and, separately, for operating surgeon. Odds ratios and confidence intervals were compared with those of the primary model to assess stability of effect estimates. All analyses were performed using PQStat v.1.8.6 (PQStat Software, Poznan, Poland) and Python (statsmodels, scipy, scikit-learn and pandas packages).

## Results

Between April 2023 and December 2024, 237 patients underwent PN at our Institution. All patients were examined for eligibility. Of 237 consecutive patients undergoing PN between April 2023 and December 2024, 200 met all inclusion criteria and had complete data for analysis (11 had recurrent tumors, 1 had a prior nephropexy, 9 had missing CTs, 4 declined consent, 12 had incomplete laboratory test results). Table 1 summarizes the baseline characteristics of the cohort. Among the 200 patients who underwent partial nephrectomy, APF was observed in 34 patients (17%). There was no significant difference in the rate of APF detection between surgical approaches (open 21.7%, laparoscopic 15.7%, robotic 13,8%, *p* = 0.381) or surgeons (surgeon 1 21.7%, surgeon 2 18.6%, surgeon 3 6.7%, *p* = 0.097).

**Table 1:** Patient and tumor-related characteristics in the whole cohort and according to presence of adherent perinephric fat

Variable	Total (n = 200)	APF (n = 34)	No APF (n = 166)	p value
Age (years), n (%)	63 (53–70)	66.5 (62–74)	62 (51–69)	**0.004**
Gender, n (%)				** < 0.001**
Female	75 (37.5)	2 (5.9)	73 (44)	
Male	125 (62.5)	32 (94.1)	93 (56)	
BMI (kg/m^2^)	27.8 (25.4–31.2)	29.7 (26.9–31.3)	27.5 (24.9–31)	**0.012**
Waist circumference (cm)	104 (97.7–111)	110 (103–117)	103 (97–110)	** < 0.001**
Hypertension, n (%)				** < 0.001**
No	67 (33.5)	3 (8.8)	64 (38.5)	
Yes	133 (66.5)	31 (91.2)	102 (61.5)	
Diabetes, n (%)				0.131
No	155 (77.5)	23 (67.6)	132 (79.5)	
Yes	45 (22.5)	11 (32.4)	34 (20.5)	
Hypolipidemic agents use, n (%)				0.058
No	139 (69.5)	19 (55.9)	120 (72.3)	
Yes	61 (30.5)	15 (44.1)	46 (27.7)	
Metabolic syndrome, n (%)				**0.027**
No	81 (40.5)	8 (23.5)	73 (44)	
Yes	119 (59.5)	26 (76.5)	93 (56)	
Dyslipidemia, n (%)				0.582
No	79 (39.5)	12 (35.3)	67 (40.4)	
Yes	121 (60.5)	22 (64.7)	99 (59.6)	
CCI	5 (3–6)	6 (5–7)	5 (3–6)	**0.001**
History of smoking, n (%)				0.897
No	143 (71.5)	24 (70.6)	119 (71.7)	
Yes	57 (28.5)	10 (29.4)	47 (28.3)	
Albumin (g/dl)	4.66 (4.5–4.82)	4.57 (4.29–4.75)	4.67 (4.52–4.83)	**0.037**
Total protein (g/dl)	7.36 (7.08–7.64)	7.27 (7–7.59)	7.38 (7.13–7.66)	0.235
ALT (IU/L)	24 (18–34)	25 (18–30)	24 (18–33)	0.513
AST (IU/L)	24 (20–28)	23 (18–30)	24 (20–28)	0.595
GGTP (U/L)	25 (17–39)	27.5 (21–39.8)	24 (16–38.8)	0.26
ALP (U/L)	74.5 (59–90.7)	80.5 (67.8–95.5)	70.4 (58–90)	0.185
LDH (U/L)	186 (164–214)	189 (161–215)	186 (165–212)	0.84
Bilirubin (mg/dl)	0.42 (0.29–0.56)	0.49 (0.34–0.66)	0.42 (0.29–0.55)	0.16
CRP (mg/dl)	0.13 (0.1–0.25)	0.15 (0.1–0.5)	0.13 (0.1–0.23)	0.064
Total cholesterol (mg/dl)	194 (163–227)	186 (149–211)	196 (168–232)	**0.034**
LDL-C (mg/dl)	102.6 (78.1–128.4)	101.6 (73.7–126)	102.8 (79.6–128.5)	0.604
HDL-C (mg/dl)	52.9 (43.9–66.4)	46.8 (41.6–52.1)	54.7 (44.1–68.8)	** < 0.001**
Triglycerides (mg/dl)	144 (102–206)	142 (117–191)	146 (102–207)	0.956
Ferritin (ng/ml)	102.4 (57.8–223.2)	146.6 (67.6–295.4)	99.4 (57.6–211.3)	**0.044**
Fasting glucose (mg/dl)	99 (91–111)	107.5 (93.3–116.8)	98.5 (90.3–109.8)	**0.044**
Preoperative creatinine (mg/dl)	0.83 (0.72–1.02)	1.03 (0.86–1.19)	0.81 (0.7–0.95)	** < 0.001**
Preoperative eGFR (ml/min/1,73m^2^)	93.8 (80–102.7)	76.3 (64.9–99.1)	95.2 (83–103.6)	** < 0.001**
Urea (mg/dl)	34.7 (28.9–41.7)	39.1 (34.9–49.3)	33.1 (27.9–40.7)	** < 0.001**
Maximal tumor diameter (mm)	31 (21–45)	35 (21.3–49)	30 (20.3–44)	0.4
RENAL nephrometry score, n (%)				0.218
4–6	70 (35)	10 (29)	60 (36)	
7–9	98 (49)	21 (62)	77 (46)	
10–12	32 (16)	3 (9)	29 (18)	
Posterior perinephric fat thickness (mm)	19 (7–27)	31 (25–37)	14 (7–24)	** < 0.001**
Stranding, n (%)				** < 0.001**
None	111 (55.5)	2 (5.9)	109 (65.7)	
Type 1	66 (33)	22 (64.7)	44 (26.5)	
Type 2	23 (11.5)	10 (29.4)	13 (7.8)	
Surgical approach, n (%)				0.381
Open	69 (34.5)	15 (44.1)	54 (32.5)	
Laparoscopic	51 (25.5)	8 (23.5)	43 (25.9)	
Robotic	80 (40)	11 (32.4)	69 (41.6)	
Surgeon, n (%)				0.097
Surgeon 1	69 (34.5)	15 (44.1)	54 (32.5)	
Surgeon 2	86 (43)	16 (47.1)	70 (42.2)	
Surgeon 3	45 (22.5)	3 (8.8)	42 (25.3)	
Tumor histology, n (%)				0.086
ccRCC	113 (56.5)	25 (73.5)	88 (53)	
chRCC	11 (5.5)	0 (0)	11 (6.6)	
pRCC type I and II	30 (15)	5 (14.7)	25 (15)	
Benign	46 (23)	4 (11.8)	42 (25.3)	

Bolded numbers indicate statistically significant results

Data are shown as number (percentage). For continuous variables, the median and interquartile range is given

*ALP* alkaline phosphatase, *ALT* alanine aminotransferase, *APF* adherent perinephric fat, *AST* aspartate transaminase, *BMI* body mass index, *CCI* Charlson Comorbidity Index, *ccRCC* clear cell renal cell carcinoma, *chRCC* chromophobe renal cell carcinoma, *CRP* C-reactive protein, *eGFR* estimated glomerular filtration rate, *GGTP* gamma-glutamyltransferase, *HDL-C* high density lipoprotein cholesterol, *LDH* lactate dehydrogenase, *LDL-C* low density lipoprotein cholesterol, *pRCC* papillary renal cell carcinoma, *TG* triglycerides

The median age was 63 years (IQR: 53–70); most of the patients were male (62.5%) with a median BMI of 27.8 kg/m^2^ (IQR: 25.4–31.2). The median tumor diameter was 31 mm (IQR: 21–45); 113 (56.5%) tumors were clear cell renal cell carcinoma (RCC), 11 (5.5%) were chromophobe RCC, 30 (15%) were papillary RCC type I and II, and the remaining 46 (23%) had benign histology.

Compared with patients without APF, those with APF were significantly older (66.5 vs. 63 years, *p* = 0.004), more often male (94% vs. 56%, *p* < 0.001), and had higher rates of metabolic syndrome (76.5% vs. 56%, *p* = 0.029) and hypertension (91% vs. 61%, *p* =  < 0.001). They also exhibited higher BMI (27.7 vs. 27.5 kg/m^2^, *p* = 0.012), larger waist circumference (110 vs. 103mm, *p* =  < 0.001) and greater comorbidity burden (CCI 6 vs. 5, *p* = 0.001). Liver enzymes (ALT, AST, GGTP, ALP, LDH) and bilirubin did not differ between the groups. Patients with APF had higher levels of ferritin (146.6 vs. 99.4 ng/ml, *p* = 0.044), fasting glucose (107.5 vs. 98.5 mg/dl, *p* = 0.044), preoperative creatinine (1.03 vs. 0.81 mg/dl, *p* =  < 0.001), urea (39.1 vs. 33.1 mg/dl, *p* =  < 0.001) and lower levels of albumin (4.57 vs. 4.67 g/dl, *p* = 0.037), TC (186 vs. 196 mg/dl, *p* = 0.034) and HDL-C (46.8 vs. 54.7 mg/dl, *p* =  < 0.001). On preoperative CT scans they exhibited greater perinephric fat thickness (31 vs. 14 mm, *p* < 0.001) and more frequent perinephric stranding (type 1 64.7% vs. 26.5%, type 2 29.4 vs. 7.8%, *p* < 0.001). Tumor histology did not differ between groups (*p* = 0.086).

After determination of optimal cut-off values for the continuous variables using Youden index, on univariate logistic regression analysis all variables remained statistically significant, except for ferritin (*p* = 0.07) (Table 2). As stranding type 1 and 2 were characterized by comparable odds ratios (5.08 and 4.9, respectively), we decided to join the two variables creating a new variable (stranding none/present) as presented in Table 2. On multivariable analysis using stepwise regression, presence of stranding, high posterior perinephric fat thickness and urea and low albumin and HDL-C were identified as independent predictors of APF (Table 3). The model demonstrated very good discrimination with an AUC of 0.92 (95% confidence interval 0.88–0.96). Internal validation using 1,000 bootstrap resamples demonstrated minimal optimism (mean optimism 0.001), yielding an optimism-corrected AUC of 0.91. Calibration was assessed using a calibration curve based on deciles of predicted risk, demonstrating good agreement between predicted and observed probabilities across the range of risk (Supplementary Figure S1). In sensitivity analysis using continuous predictors, posterior perinephric fat thickness remained significantly associated with APF presence, while urea, HDL-C, and albumin did not reach statistical significance as continuous variables (Supplementary Table S2). On that basis, a risk score named SHARP-U score (acronym from Stranding, HDL-C, Albumin, Renal Perinephric fat thickness, Urea) for the prediction of APF presence was created. To create a clinically practical scoring system, regression coefficients were transformed into integer point values based on the relative magnitude of the odds ratios. Predictors with odds ratios ≥ 9 were assigned 2 points, whereas predictors with odds ratios between 3 and 7 were assigned 1 point as presented in Table 3. The observed proportions of patients with APF and the predicted probabilities of APF according to SHARP-U score are presented in Table 4. Table 5 summarizes the SHARP-U scoring system. Sensitivity analyses demonstrated that adjustment for surgical approach or operating surgeon did not substantially alter the associations between SHARP-U predictors and the presence of APF (Supplementary Table S3). The optimal cut-off, determined by the highest Youden index (0.69), was identified at a SHARP-U score ≥ 4, yielding a sensitivity of 97.1%, specificity of 71.7%, positive predictive value (PPV) of 41.3%, and negative predictive value (NPV) of 99.2%.

**Table 2:** Univariable logistic regression analyses of predictors of adherent perinephric fat

Variable	Odds ratio (95% CI)	p value
Age ≥ 62 years	3.17 (1.36–7.41)	**0.004**
Gender		
Female	Reference	
Male	12.55 (2.91–54.14)	** < 0.001**
BMI ≥ 26.5 kg/m^2^	4.16 (1.53–11.31)	**0.002**
Waist circumference ≥ 110 cm	3.40 (1.6–7.27)	**0.001**
Hypertension		
No	Reference	
Yes	6.48 (1.9–22.08)	** < 0.001**
Metabolic syndrome		
No	Reference	
Yes	2.74 (1.13–6.65)	**0.017**
CCI ≥ 5	3.02 (1.29–7.07)	**0.002**
Albumin ≤ 4.3 g/dl	3.37 (1.35–8.47)	**0.013**
Total cholesterol ≤ 225 mg/dl	3.05 (1.02–9.13)	**0.026**
HDL-C ≤ 53 mg/dl	4.04 (1.73–9.45)	** < 0.001**
Ferritin ≥ 170 mg/dl	2.0 (0.9–4.2)	0.07
Fasting glucose ≥ 106 mg/dl	2.9 (1,4–6.2)	**0.004**
Preoperative creatinine ≥ 0,92 mg/dl	6.83 (2.97–15.7)	** < 0.001**
Preoperative eGFR ≤ 87 ml/min/1,73m^2^	4.02 (1.85–8.73)	** < 0.001**
Urea ≥ 33 mg/dl	4.56 (1.79–11.58)	** < 0.001**
Posterior perinephric fat thickness ≥ 25 mm	16.85 (6.48–43.83)	** < 0.001**
Stranding		
None	Reference	
Type 1	5.08 (2.32–11.12)	** < 0.001**
Type 2	4.90 (1.93–12.42)	**0.001**
Stranding		
None	Reference	
Present (type 1 + 2)	30.60 (7.08–132.28)	** < 0.001**

Bolded numbers indicate statistically significant results

*BMI* body mass index, *CCI* Charlson Comorbidity Index, *CI* confidence interval, *eGFR* estimated glomerular filtration rate, *HDL-C* high density lipoprotein

**Table 3:** Multivariable logistic regression analyses of predictors of adherent perinephric fat and point assignment for the SHARP-U score

Multivariable model (AUC = 0.92, 95% CI 0.88–0.96)			SHARP-U score points
Variable	OR (95% CI)	p value	
Posterior perinephric fat thickness ≥ 25 mm	9.87 (2.93–33.30)	< 0.001	2
Stranding			
None	Reference		
Present (type 1 + 2)	11.15 (2.26–55.02)	0.003	2
Urea ≥ 33 mg/dl	6.26 (1.79–21.87)	0.004	1
Albumin ≤ 4.3 g/dl	6.90 (1.71–21.78)	0.007	1
HDL-C ≤ 53 mg/dl	3.60 (1.23–10.57)	0.019	1

*AUC* area under the receiver operating characteristics curve, *CI* confidence interval, *HDL-C* high density lipoprotein, *OR* odds ratio

**Table 4:** Observed proportions of patients with adherent perinephric fat (APF) and the predicted probabilities of APF according to SHARP-U score

SHARP-U score	Proportion of patients with APF	Predicted probability of APF (actual) (95% CI)	Predicted probability of APF (logistic regression) (95% CI)
0	0/40	0.0% (0.0–8.8)	0.1% (0.0–1.2)
1	0/38	0.0% (0.0–9.2)	0.4% (0.1–4.2)
2	0/25	0.0% (0.0–13.3)	1.5% (0.2–14.2)
3	1/17	5.9% (1.0–27)	5.0% (0.4–40.3)
4	6/25	24% (11.5–43.4)	15.0% (1.1–74.4)
5	9/20	45% (25.8–65.8)	37.3% (2.6–92.9)
6	15/23	65.2% (44.9–81.2)	66.7% (6.2–98.4)
7	3/3	100% (43.9–100)	87.1% (13.7–99.7)

*APF* adherent perinephric fat, *CI* confidence interval

**Table 5:** SHARP-U score for preoperative prediction of adherent perinephric fat

Predictor	Definition	Points
Posterior perinephric fat thickness	≥ 25 mm on preoperative CT	2
Perinephric fat stranding	Present on preoperative CT	2
Serum urea	≥ 33 mg/dl	1
Serum albumin	≤ 4.3 g/dl	1
HDL cholesterol	≤ 53 mg/dl	1
Total SHARP-U score	Sum of all points	0–7

Posterior perinephric fat thickness measurement and perinephric fat stranding assessment are shown in Fig. 1. and Fig. 2. SHARP-U score is calculated by summing the points assigned to each predictor present. Higher scores indicate an increased probability of adherent perinephric fat

*CT* computed tomography

## Discussion

The presence of APF during PN represents a well-recognized source of technical difficulty, often necessitating subcapsular dissection to safely separate densely adherent adipose tissue from the renal capsule while avoiding bleeding and tumor injury [1]. Accumulating evidence suggests that APF is associated with longer operative times, increased blood loss, and greater intraoperative complexity [5]. Although the impact of APF on postoperative outcomes remains debated [14], there is broad consensus that its intraoperative presence substantially increases surgical challenge and may compromise procedural efficiency and safety.

Previous research has largely focused on clinical and radiological predictors of APF, with the Mayo Adhesive Probability score being the most cited tool [8]. However, these models do not account for metabolic or inflammatory parameters, despite increasing evidence suggesting a link between systemic inflammation, metabolic dysregulation, and perinephric fibrosis. In the present study, we prospectively evaluated a contemporary cohort of patients undergoing partial nephrectomy across open, laparoscopic, and robotic approaches and identified elevated urea, reduced serum albumin and low HDL-C as novel independent predictors of APF. On that basis, we developed the SHARP-U score, a simple and pragmatic preoperative risk stratification tool that allows clinicians to estimate the likelihood of encountering APF using routinely available imaging and laboratory data. The SHARP-U score expands upon prior models by integrating routinely available laboratory parameters, thereby capturing a broader biological context that may influence APF development.

APF was observed in 17% of patients undergoing PN, consistent with the wide range reported in prior studies (10.6–55.2%) [3, 4]. Our findings confirm and expand on prior evidence that APF is associated with male sex, older age, higher BMI, hypertension and the presence of metabolic syndrome [5, 7]. In contrast to the study by Borregales et al., diabetes was not a risk factor for APF [9].

Among radiological features, perinephric fat thickness and stranding emerged as the strongest predictors of APF, consistent with earlier imaging studies [8, 9]. Whereas prior work suggested that type 2 stranding confers the highest risk of APF, we observed comparable odds ratios for stranding types 1 and 2. This discrepancy may reflect the partially subjective nature of the original grading system proposed by Davidiuk et al.[8]. To reduce interobserver variability and enhance clinical applicability, we combined stranding types into a dichotomous variable (absent vs present), which demonstrated strong independent predictive value in the multivariable model (OR = 11.15).

Unlike stranding, which may be subjectively interpreted, laboratory parameters offer standardized and reproducible metrics. The association of albumin, urea, and HDL-C with APF highlights the likely contribution of systemic metabolic and inflammatory processes to the pathogenesis of APF. Hypoalbuminemia and low HDL-C are well-established markers of chronic low-grade inflammation and metabolic dysfunction [15–17], while elevated urea may reflect renal impairment, catabolic stress, or dehydration [18]. When analyzed as continuous variables in sensitivity analysis, urea, HDL-C, and albumin did not retain statistical significance. This likely reflects non-linear or threshold-dependent relationships with APF, as well as limited statistical power given the relatively small number of APF events. Importantly, these variables demonstrated independent associations when dichotomized and contributed to the strong overall discriminatory performance of the SHARP-U score.

Rather than acting as direct causal drivers, these parameters likely serve as surrogate markers of an adverse metabolic–inflammatory milieu, which may predispose to adipose tissue dysfunction and fibrotic remodeling. This interpretation is supported by the observed association between APF and metabolic syndrome, a condition characterized by visceral adiposity, dyslipidemia, insulin resistance, and chronic inflammation, which has previously been identified as an independent risk factor for APF [7]. While mechanistic pathways cannot be inferred from this study, these findings support the concept that APF represents a localized manifestation of broader metabolic and inflammatory processes and can be considered hypothesis-generating for further studies.

The presented SHARP-U score demonstrated high sensitivity of 97.1% and NPV of 99.2% with a modest PPV of 41.3%. Notably, patients with a score of 0–2 had virtually no risk of APF, while those with a score of 6–7 had a ≥ 65% observed incidence. The performance characteristics of SHARP-U indicate that it is best suited as a rule-out and surgical planning tool rather than a diagnostic test for APF. The high sensitivity and negative predictive value allow reliable identification of patients at very low risk of APF, in whom standard operative planning is appropriate. Conversely, a high SHARP-U score should not be interpreted as definitive evidence of APF, but rather as an indicator of increased likelihood of surgical complexity. In previous studies APF has been consistently associated with adverse perioperative outcomes, including longer operative times, increased blood loss, and higher transfusion rates [5]. In clinical practice, the SHARP-U score may support preoperative planning by informing selection of surgical expertise, anticipation of operative time and blood loss, choice of surgical approach or access route, and patient counseling regarding procedural difficulty and perioperative risk. The question of whether preoperative identification of APF should influence treatment decisions has not yet been answered. Future studies should focus on defining when to opt for ablative techniques and, if surgery is chosen, whether to modify the surgical approach (e.g., access route, extent, instrumentation). Additionally, it remains to be established whether preoperative optimization of metabolic parameters could mitigate APF severity. We feel that APF anticipation should not be the sole determinant of treatment modification, but rather serve as an adjunct to established anatomic classification systems.

Several limitations merit consideration. First, APF was defined intraoperatively based on surgeon assessment, which is inherently subjective. While we attempted to reduce interobserver variability by using a consistent definition requiring subcapsular dissection and limiting the number of surgeons, this remains a source of potential bias. Inter-reader agreement could not be calculated, as APF evaluation was performed by a single reader. The inclusion of multiple surgical approaches may have further confounded intraoperative APF assessment. However, the rates of APF detection did not significantly differ between surgeons and surgical approaches. Sensitivity analyses adjusting for surgical approach and, separately, for operating surgeon demonstrated stable effect estimates for all SHARP-U predictors, supporting the robustness of the model. Nonetheless, residual confounding cannot be entirely excluded and should be addressed in future multicenter validation studies. Second, the cut-offs used in the SHARP-U score were derived from the study cohort and may not directly translate to other settings. Finally, this study was conducted at a single high-volume tertiary center, which may limit generalizability to lower-volume institutions or centers with differing patient populations and surgical practices. As such, the SHARP-U score should be considered exploratory and hypothesis-generating until externally validated. External validation in independent, preferably multicenter, prospective cohorts is essential to confirm discrimination, calibration, and clinical utility. A temporal validation cohort or registry-based validation would be particularly valuable to assess performance across evolving surgical techniques.

Despite these limitations, this study identifies novel metabolic predictors of APF and introduces a clinically accessible risk stratification tool. The SHARP-U score may assist urologists in anticipating surgical difficulty, optimizing perioperative planning, and improving patient counseling. External prospective validation—ideally in multicenter cohorts or temporal validation studies—is required to confirm its robustness, calibration, and generalizability. Future research should also explore whether preoperative identification of APF should influence treatment decisions to improve surgical outcomes.

## Supplementary Information

Below is the link to the electronic supplementary material.

Supplementary file1 (DOCX 50 KB)

## Data Availability

The data that support the findings of this study are available from the corresponding author upon reasonable request.
