# Supplementary material for: Metabolic insights and novel risk score for adherent perinephric fat in partial nephrectomy: results from a prospective study
Source: Int Urol Nephrol. 2026 Feb 6;58(8):2973–81. doi: 10.1007/s11255-026-05031-5 (PMC13375828; doi:10.1007/s11255-026-05031-5)
Supplement: Supplementary file 1 — Supplementary file1 (DOCX 50 KB) [file 11255_2026_5031_MOESM1_ESM.docx]

Supplementary Figure S1. Calibration curve of observed versus predicted probability of adherent perinephric fat by the SHARP-U score


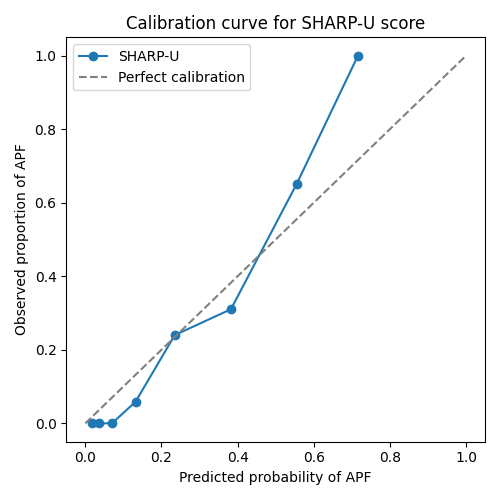


Supplementary Table S2. Multivariable logistic regression model using continuous predictors for APF prediction

| Variable | Standardized coefficient | OR (95% CI) | p value |
| --- | --- | --- | --- |
| Posterior perinephric fat thickness | 1.18 | 1.10 (1.04-1.15) | 0.001 |
| Stranding |  |  |  |
| None | Reference | Reference |  |
| Present (type 1+2) | -2.99 | 12.53 (2.42-64.78) | 0.003 |
| Urea | 2.53 | 1.03 (0.99-1.08) | 0.13 |
| Albumin | -2.7 | 0.34 (0.08-1.46) | 0.147 |
| HDL-C | 0.39 | 0.98 (0.95-1.02) | 0.412 |

AUC = area under the receiver operating characteristics curve; CI = confidence interval; HDL-C = high density lipoprotein cholesterol; OR = odds ratio

Supplementary Table S3. Sensitivity analyses of the multivariable logistic regression model for prediction of adherent perinephric fat, adjusted for surgical approach and operating surgeon.

| Predictor | Primary Model OR (95% CI) | p value | Model adjusted for surgical approach OR (95% CI) | p value | Model adjusted for surgeon OR (95% CI) | p value |
| --- | --- | --- | --- | --- | --- | --- |
| Posterior perinephric fat thickness ≥25 mm | 9.87 (2.93-33.3) | <0.001 | 9.49 (2.81-32.09) | <0.001 | 9.39 (2.75-32.05) | <0.001 |
| Perinephric fat stranding (present) | 11.15 (2.26-55.02) | 0.003 | 11.32 (2.25-56.96) | 0.003 | 10.41 (2.07-52.40) | 0.004 |
| Urea ≥33 mg/dl | 6.26 (1.79-21.87) | 0.004 | 6.04 (1.72-21.22) | 0.005 | 6.49 (1.82-23.23) | 0.004 |
| Albumin ≤4.3 g/dl | 6.9 (1.71-21.78) | 0.007 | 6.50 (1.59-26.65) | 0.009 | 6.06 (1.46-25.06) | 0.013 |
| HDL-C ≤53 mg/dl | 3.6 (1.23-10.57) | 0.019 | 3.61 (1.22-10.68) | 0.02 | 3.64 (1.23-10.72) | 0.019 |
| Surgical approach (reference: open) | — | — | Reference |  | — | — |
| Laparoscopic | — | — | 0.62 (0.18-2.18) | 0.457 | — | — |
| Robotic | — | — | 0.71 (0.22-2.23) | 0.554 | — | — |
| Surgeon (reference: Surgeon 1) | — | — | — | — | Reference |  |
| Surgeon 2 | — | — | — | — | 0.82 (0.28-2.39) | 0.709 |
| Surgeon 3 | — | — | — | — | 0.36 (0.07-1.78) | 0.211 |

CI = confidence interval; HDL-C = high density lipoprotein cholesterol; OR = odds ratio
